# Enhanced biliary cannulation and sphincterotomy using a novel rotatable sphincterotome for surgically altered anatomy

**DOI:** 10.1055/a-2501-3103

**Published:** 2025-01-14

**Authors:** Yasuhiro Kuraishi, Yuki Shimizu, Akira Nakamura, Takeji Umemura

**Affiliations:** 1 Department of Gastroenterology, Shinshu University Hospital, Matsumoto, Japan

Balloon enteroscopy-assisted endoscopic retrograde cholangiopancreatography (BE-ERCP) is effective in patients with surgically altered anatomy. However, challenges remain when performing BE-ERCP owing to limited scope and device maneuverability 1 2 ; the scope often aligns tangentially to the papilla, complicating selective biliary cannulation. Meanwhile, conventional sphincterotomes are unsuitable for performing sphincterotomy as their blades misalign with bile duct direction. A newly developed sphincterotome (Engetsu; Kaneka Medix, Osaka, Japan) features 360-degree rotational capability and adjustable blade mobility for extension and shortening ( Fig. 1 ). We herein describe a successful case using this rotatable sphincterotome for biliary canulation and sphincterotomy in BE-ERCP.

**Fig. 1:**
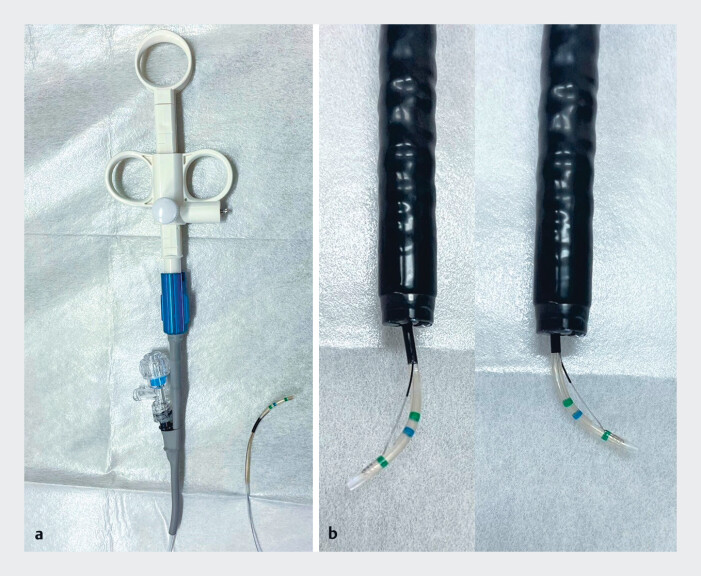
The newly developed sphincterotome (Engetsu; Kaneka Medix, Osaka, Japan). **a, b** The device features 360-degree rotational capability and adjustable blade mobility for extension and shortening, enabling precise biliary cannulation and safe sphincterotomy in patients with surgically altered anatomy.

A 71-year-old man presented with obstructive jaundice appearing as distal cholangiocarcinoma on computed tomography ( Fig. 2 ). He had previously undergone gastrojejunostomy for duodenal stenosis secondary to an ulcer. We performed ERCP using a short-type single-balloon enteroscope (SIF-H290; Olympus Medical Systems, Tokyo, Japan) ( Fig. 3 , Video 1 ). Initial attempts at biliary cannulation with a conventional ERCP catheter were unsuccessful due to the enlarged, protruding duodenal papilla and tangential scope orientation, which caused the catheter to misalign with the bile duct. We switched to the Engetsu for biliary cannulation.

**Fig. 2:**
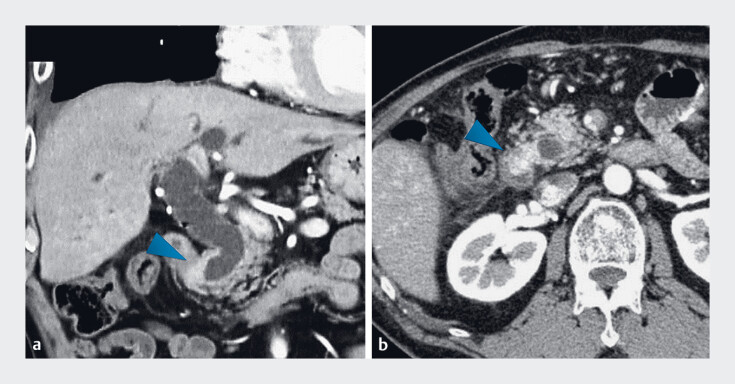
Computed tomography depicting a tumor in the distal bile duct (arrowheads). The images revealed stricture at the tumor site accompanied by proximal bile duct dilation. **a** Coronal plane. **b** Axial plane.

**Fig. 3:**
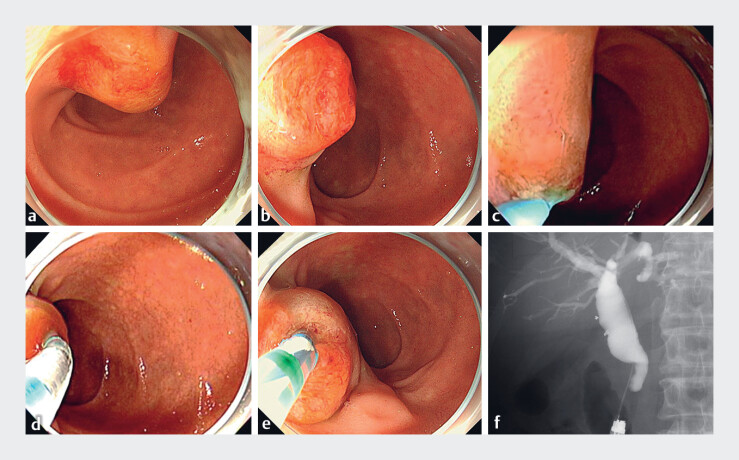
Biliary cannulation. **a, b** Endoscopic appearance of the duodenal papilla, displaying marked enlargement and protruding papilla. **c** Initial attempts at biliary cannulation with a conventional straight catheter failed as the scope became positioned tangentially to the papilla, causing the catheter to misalign with the bile duct. **d, e** The Engetsu sphincterotome (Kaneka Medix, Osaka, Japan) was used for biliary cannulation. By precisely adjusting its rotation and angulation, it could be aligned with the bile duct axis, facilitating successful cannulation. **f** Cholangiography showed distal bile duct stricture with proximal dilation.

A novel rotatable sphincterotome effectively addressed the challenges of endoscopic retrograde cholangiopancreatography in a patient with surgically altered anatomy, facilitating precise biliary cannulation and safe sphincterotomy.Video 1

By adjusting its rotation and angulation, the sphincterotome could be aligned with the bile duct axis, enabling successful cannulation. Cholangiography showed distal bile duct stricture, and intraductal ultrasonography revealed extensive upstream bile duct wall thickening contiguous with the distal tumor. Sphincterotomy was subsequently performed using the Engetsu ( Fig. 4 ). The blade of the sphincterotome was aligned with the bile duct direction through rotation and angulation, allowing a safe and adequate incision. We next performed transpapillary biopsy and nasobiliary drainage. During the second ERCP, a cholangioscope was inserted through the post-sphincterotomy orifice to assess the horizontal tumor spread.

**Fig. 4:**
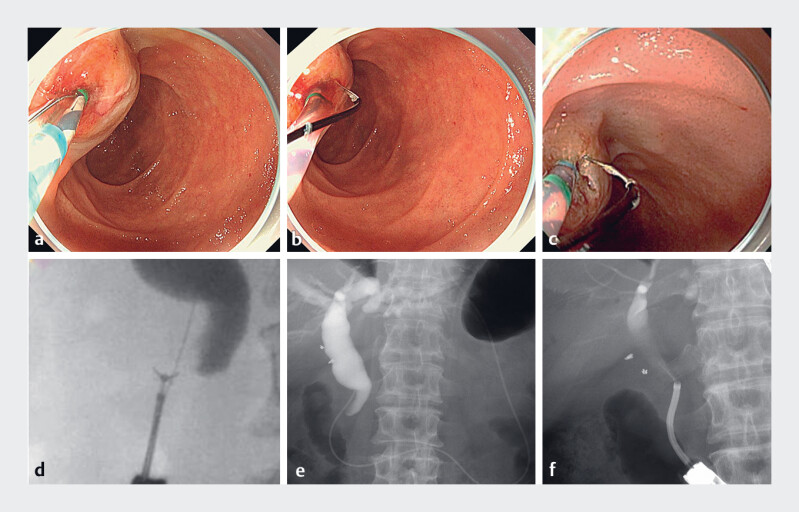
Cannulation and tumor assessment. **a** The Engetsu (Kaneka Medix, Osaka, Japan) was employed for sphincterotomy. When the device was initially advanced out of the scope, the blade was not aligned with the bile duct. **b, c** The blade of the sphincterotome was aligned with the bile duct direction through precise rotation and angulation, enabling a safe and adequate incision. **d** Transpapillary biopsy was performed on the stricture of the distal bile duct. **e** A nasobiliary drainage tube was temporarily placed. **f** During the second session, a cholangioscope was inserted through the post-sphincterotomy orifice to assess the extent of horizontal tumor spread.

The Engetsu sphincterotome effectively addressed BE-ERCP-related challenges by facilitating precise biliary cannulation and safe sphincterotomy, highlighting its potential to improve procedural success in complex ERCP procedures.

Endoscopy_UCTN_Code_TTT_1AR_2AK

## Notes

Endoscopy E-Videos https://eref.thieme.de/e-videos
*E-Videos* is an open access online section of the journal *Endoscopy* , reporting on interesting cases and new techniques in gastroenterological endoscopy. All papers include a high-quality video and are published with a Creative Commons CC-BY license. Endoscopy E-Videos qualify for HINARI discounts and waivers and eligibility is automatically checked during the submission process. We grant 100% waivers to articles whose corresponding authors are based in Group A countries and 50% waivers to those who are based in Group B countries as classified by Research4Life (see: https://www.research4life.org/access/eligibility/ ). This section has its own submission website at https://mc.manuscriptcentral.com/e-videos .
